# Standardised 3D-CT lung volumes for patients with idiopathic pulmonary fibrosis

**DOI:** 10.1186/s12931-022-02062-1

**Published:** 2022-06-01

**Authors:** Yuko Tanaka, Yuzo Suzuki, Hirotsugu Hasegawa, Koshi Yokomura, Atsuki Fukada, Yusuke Inoue, Hironao Hozumi, Masato Karayama, Kazuki Furuhashi, Noriyuki Enomoto, Tomoyuki Fujisawa, Yutaro Nakamura, Naoki Inui, Takafumi Suda

**Affiliations:** 1 Second Division, Department of Internal Medicine, Hamamatsu University School of Medicine, 1-20-1 Handayama Higashi-ku, Hamamatsu, Shizuoka 431-3192 Japan; 2 Department of Respiratory Medicine, Seirei Mikatahara General Hospital, 3453 Mikatahara-cho, Kita-ku, Hamamatsu, Shizuoka 433-8558 Japan

**Keywords:** Idiopathic pulmonary fibrosis, Acute exacerbation, Three-dimensional computed tomography, Lung volume

## Abstract

**Background:**

The assessment of lung physiology via pulmonary function tests (PFTs) is essential for patients with idiopathic pulmonary fibrosis (IPF). However, PFTs require active participation, which can be challenging for patients with severe respiratory failure, such as during moments of acute exacerbation (AE) of IPF. Recent advances have enabled the re-construction of 3-dimensional computed-tomography (3D-CT) images. This study established a standardisation method and quantitative analysis of lung volume (LV) based on anthropometry using 3D-CT images.

**Methods:**

This is a retrospective multi-center cohort study. The standardised 3D-CT LV in patients with IPF at diagnosis (n = 140) and during AE (cohort1; n = 61 and cohort2; n = 50) and those of controls (n = 53) were assessed.

**Results:**

The standardised 3D-CT LVs at IPF diagnosis were less than those of control patients, especially in the lower lung lobes. The standardised 3D-CT LVs were correlated with forced vital capacity (FVC) and validated using the modified Gender-Age-Physiology (GAP) index. The standardised 3D-CT LVs at IPF diagnosis were independently associated with prognosis. During AE, PFTs were difficult to perform, 3D-CT analyses revealed reduced lung capacity in both the upper and lower lobes compared to those obtained at diagnosis. Lower standardised 3D-CT LVs during AE were independently associated with worse outcomes in the two independent cohorts. In particular, volume loss in the upper lobe at AE had prognostic values.

**Conclusions:**

A novel image quantification method for assessing pulmonary physiology using standardised 3D-CT-derived LVs was developed. This method successfully predicts mortality in patients with IPF and AE of IPF, and may be a useful alternative when PFTs cannot be performed.

**Supplementary Information:**

The online version contains supplementary material available at 10.1186/s12931-022-02062-1.

## Background

Idiopathic pulmonary fibrosis (IPF) is the most common and progressive fibrosing form of interstitial lung disease (ILD). It eventually leads to respiratory failure, with a poor patient prognosis [1, 2]. Pulmonary function tests (PFTs) have been widely used in the management of patients with IPF to assess the disease severity and predict progression and prognosis. However, PFTs require active patient participation. An inability or unwillingness to follow directions during PFTs leads to submaximal test results [3]. In addition, for patients with advanced IPF and during times of acute exacerbation (AE) of IPF, it is difficult to perform PFTs due to severe respiratory failure. Therefore, an alternative method that accurately evaluates lung function in patients with severe respiratory failure is needed.

Computed tomography (CT) is essential for the diagnosis [1] and assessment of ILDs [4–7], and are therefore routinely performed in patients with ILD. CT imaging can be performed with minimal patient effort, such as a full inspiration breath-hold for a few seconds, making it possible in patients with severe respiratory failure, such as advanced IPF or AE of IPF. In addition, recent advances in three-dimensional computed tomography (3D-CT) allow for detailed reconstruction of lung images, providing accurate lung volume (LV) measurements in different lung lobes.

In this study, LVs were quantitatively analysed and standardised using reconstructed 3D-CT images of patients with IPF, and the correlation between the standardised 3D-CT LV and the results of PFTs was examined. The prognostic values of standardised 3D-CT LVs in patients with IPF, including those who developed AE, were also determined. We hypothesise that the measurement of LVs using reconstructed 3D-CT images is correlated with the results of PFT, and that these LV measurements are clinically useful as an alternative to PFTs, even in patients with severe respiratory failure who cannot perform PFTs.

## Methods

### Patients

The data from a total of 190 consecutive patients with IPF who were admitted to the Hamamatsu University School of Medicine Hospital from January 2007 to July 2020 were retrospectively reviewed. Overall, 50 patients were excluded from this study, including 33 who underwent CT with a slice thickness > 5 mm, 11 who had a history of lung resection or concomitant malignancy, and six who had insufficient clinical data regarding PFTs. Therefore, 140 patients with IPF were enrolled in this study, and 61 AEs occurred in 47 patients. To confirm the values of standardised 3D-CT LVs, another set of consecutive cohort patients with AE-IPF (n = 50) from Seirei-Mikatahara hospital were also enrolled. A total of 140 chest CTs obtained at the time of IPF diagnosis and 111 obtained during AE were analysed (Additional file 1). IPF was diagnosed based on the ATS/ERS/JRS/LATA criteria [1, 2, 8], and AEs were diagnosed based on the ATS guidelines [9, 10]. Fifty-three age- and sex-matched controls were also included in the study.

This study was approved by the Ethical Committee of Hamamatsu University School of Medicine (20-270) and was conducted in accordance with approved guidelines. The requirement of informed consent was waived due to the retrospective nature of the study.

### Quantification and standardisation of 3D-CT LVs

Chest CT images obtained at the time of IPF diagnosis and AE were retrieved from electronic medical records. All CT images were obtained for diagnostic purposes during routine clinical practice. The chest CT scans were carried out without intravenous contrast with the patient in the supine position. Patients were instructed to maintain full inspiration without coughing. SOMATOM Definition Flash (Siemens Healthcare, Tokyo, Japan) and Aquillion CX (CANON medical, Tokyo, Japan) CT scanners were used. The settings of the CT machine were as follows: detector-row configuration acquired as 128 × 0.6 mm (SOMATOM) and 64 × 0.5 mm (Aquillion CX) by double sampling, 120 kVp, and quality mAs via AEC. A sharp kernel of I70 (SOMATOM) and FC52 (Aquillion CX) were used to create 3D-CT images using 5-mm slices at 5-mm intervals in the lung parenchyma (window level: − 600 HU; window width: 1500 HU). The 3D-CT LV obtained using 5-mm slice at 5-mm intervals were significantly correlated those obtained using 0.625-mm slice at 0,625 mm intervals (r = 1.000, p < 0.0001). SYNAPSE VINCENT version 3 software (Fujifilm Medical Systems, Tokyo, Japan) was used to extract the lung fields from CT images, perform lung lobe segmentation, and analyse each LV (total lung, right lung, right upper lobe, right middle lobe, right lower lobe, left lung, left upper lobe, and left lower lobe). Digital imaging and communications in medicine data for each patient were transferred to the software anonymously. Using this software, whole lung extraction from chest CT imaging was automatically quantified by excluding the thoracic wall, mediastinum, large vessels, and airways toward the tertiary bronchi. Lung extraction and lung lobe segmentation were performed using both threshold values and anatomical knowledge-based algorithms. CT values for the whole lung were defined as between -1000 HU and 0 HU. The automatic lung extraction was assessed by a respiratory physician with eight years of experience (YT). In addition, a threshold-based volumetric CT analysis, in which threshold CT values can be flexibly determined by all users, was used. When the software failed to segment the lung or lung lobe, the respiratory physician manually segmented the lung or lung lobe to correct the problem. Thereafter, a respiratory specialist with 20 years of experience (YS) confirmed the accuracy of the segmentation results. The standardised 3D-CT LV (%) was calculated by dividing by the 3D-CT LV by the predicted forced vital capacity (FVC-predicted). The reference values of FVC (FVC-predicted) were defined based on age, sex, height, and ethnicity [11, 12].

### Data collection

Clinical characteristics at the time of IPF diagnosis (age, sex, physical examination, smoking history, blood test results, and PFT results) were retrieved from the patients’ medical records. Clinical data recorded at the time of AE were also retrieved.

### The gender-age-physiology (GAP) index

The Gender-Age-Physiology (GAP) index was assessed on the basis of data at the time of IPF diagnosis, as previously described [13]: sex (female, 0 points; male, 1 point), age (≤ 60 years , 0 points; 61–65 years, 1 point; > 65 years, 2 points), FVC (%) (> 75% , 0 points; 50–75%, 1 point; < 50% , 2 points), and %DLCO (> 55% , 0 points; 36–55%, 1 point; ≤ 35% , 2 points; cannot perform, 3 points). The GAP index was defined based on the total GAP score: stage I (0–3 points), stage II (4–5 points), and stage III (6–8 points). The modified GAP index was also evaluated using the standardised 3D-CT LV (%) in place of the FVC (%). Standardised 3D-CT LVs (%) corresponding to FVCs (%) were determined using correlation analyses and used to calculate the modified GAP index.

### Statistics

Categorical variables are presented as number (percentage) and continuous variables as mean or median (interquartile range [IQR]). The Mann–Whitney U test and Fisher’s exact test were used to compare continuous and categorical variables, respectively. Pearson’s correlation coefficient was used to determine the relationship between FVC and standardised 3D-CT LV at the time of IPF diagnosis. The overall survival time was measured from the dates of IPF diagnosis and AE diagnosis. Univariate and multivariate analyses were conducted using the Cox proportional hazards regression model. The cut-off values for standardised 3D-CT LV at the time of IPF diagnosis and AE were assessed by the median. Cumulative survival probabilities were estimated using the Kaplan–Meier method and log-rank test. The discrimination performance of the model was evaluated using concordance statistics (C-index). All statistical analyses were carried out using EZR version 1.42 software [14]. All analyses were two-tailed, and statistical significance was set to p < 0.05.

## Results

### Clinical characteristics at the time of IPF diagnosis

The median age at the time of IPF diagnosis was 70 years (IQR: 65–75 years) (Table 1). Approximately 80% of the patients were men and had a history of smoking. Most of the patients had normal to mild restrictive respiratory impairment. The median FVC was 74.4% (IQR: 65.2–86.4%) and the median decreased lung diffusion capacity for carbon monoxide (DLCO) was 64.1% (IQR: 53.0–81.7%). The GAP index was calculated for 97 patients, of which 58.8% were stage I, 27.8% were stage II, and 13.4% were stage III.

**Table 1:** Clinical characteristics of patients with IPF at diagnosis

	IPF at diagnosis (n = 140)
Age, year	70 [65–75]
Sex, male/female	119 (85.0%)/21 (15.0%)
cIPF/UIP/IPF	106 (75.7%)/34 (24.3%)
Observation period, years	3.7 [1.6–6.1]
Never smoker	27 (19.3%)
Former or current smoker	113 (80.7%)
Height, cm	161.4 [155.8–165.7]
Weight, kg	60.1 [52.7–67.1]
BMI, kg/m^2^	23.1 [21.3–25.1]
Pulmonary function test	
FVC, % predicted	74.4 [65.2–86.4]
FEV_1_/FVC, %	83.0 [79.5–88.7]
DLCO, %	64.1 [53.0–81.7] (n = 93*)
TLC, %	78.0 [67.2–87.7] (n = 93)
GAP index (stage I/II/III)	57(58.8%)/27 (27.8%)/13(13.4%) (n = 97*)
Laboratory	
PaO_2_, Torr	80.0 [71.0–91.0]
KL-6, U/ml	893 [633–1303]
SP-D, ng/ml	216 [142–347]
CRP, mg/dL	0.17 [0.07–0.39]

*IPF* idiopathic pulmonary fibrosis, *UIP* usual interstitial pneumonia, *BMI* body mass index, *FVC* forced vital capacity, *FEV*_*1.0*_ forced expiratory volume in 1.0 s, *DLCO* diffuse capacity of the lung for carbon monoxide, *GAP* gender-age-physiology, *KL-6* Krebs von den Lunge-6, *SP-D* surfactant protein-D, *CRP* C-reactive protein

*DLCO could not be measured in four patients due to dyspnea

### Standardised 3D-CT LV and its correlation with lung physiology

**Fig. 1:**
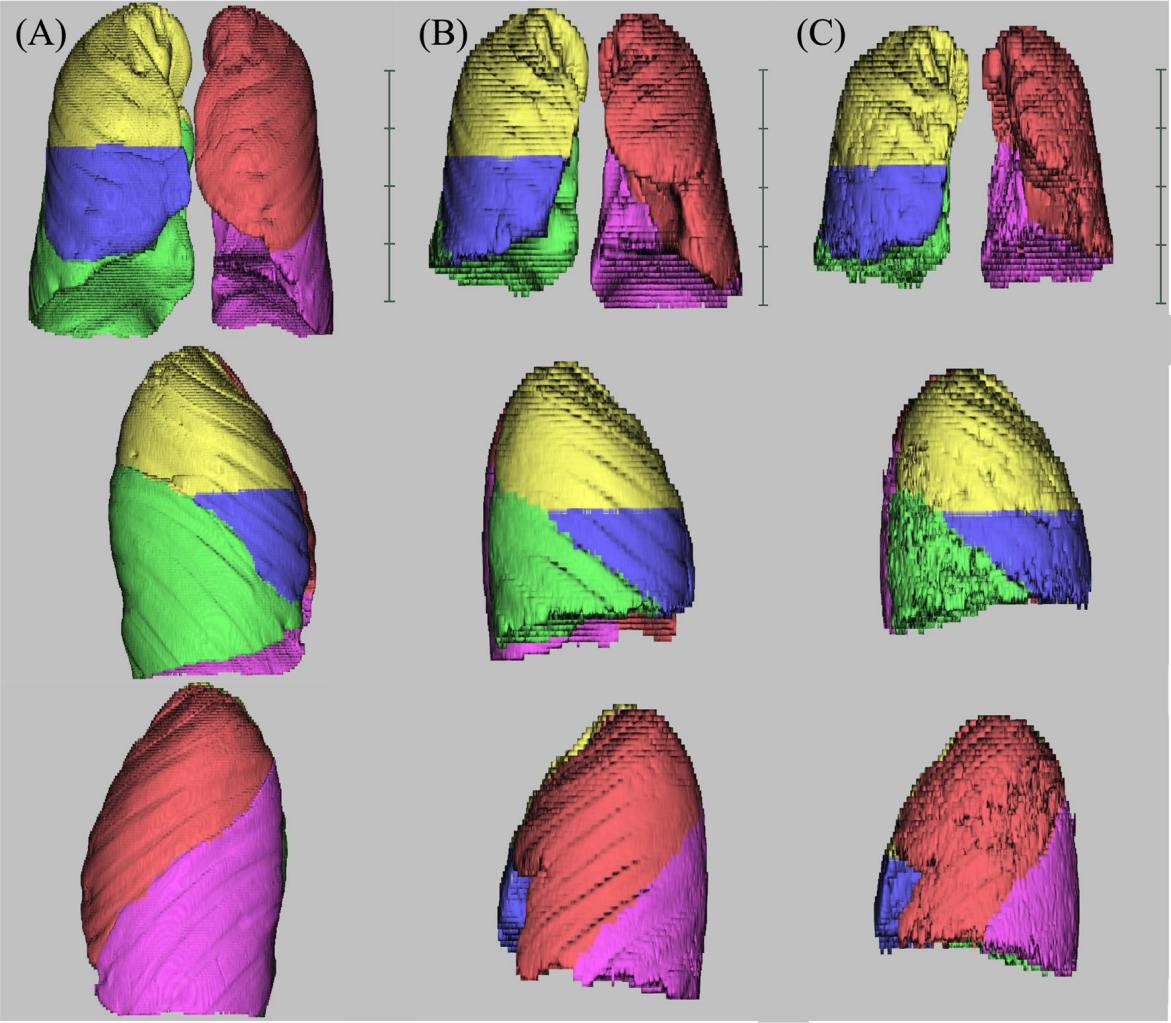
Three-dimensional lung images obtained at diagnosis and during acute exacerbations. Representative segmental lung images of age- and sex-matched control patients (**A** man, height 164.5 cm; weight 62.8 kg) and patients with IPF at the time of diagnosis (**B** man, height 167.1 cm; weight 64.1 kg) and acute exacerbation (AE) of IPF (**C** 5 years after diagnosis, height 167.4 cm; weight 62.0 kg) are represented. The same patient is shown in **B** and **C**. The lungs are color-coded as follows: right upper lobe, yellow; right middle lobe, blue; right lower lobe, green; left upper lobe, red; and left lower lobe, pink

Representative segmental lung images at IPF diagnosis and during AE and those of age- and sex-matched control patients are presented in Fig. 1. The standardisation of 3D-CT LV to FVC-predictive values is shown in Table 2. In patients with IPF, the median standardised 3D-CT total LV was 108.2% (IQR: 90.5–123.5), the standardised upper lobe LV was 52.2% (IQR: 47.9–61.4), and the standardised lower lobe LV was 39.6% (IQR: 32.4–49.0). The median standardised 3D-CT LV of the total lung and each lobe, except for the right middle lobe, was significantly lower in at diagnosis patients with IPF than in the control group. The difference in standardised 3D-CT LV between patients with IPF and the control group was more significant in the lower lobes than in the upper lobes.

**Table 2:** Comparison of standardised 3D-CT LV in patients with IPF at diagnosis and controls

Standardised 3D-CT LV, %	Controls (n = 53)	IPF at diagnosis (n = 140)	p-value
Total lung	141.3 [126.7–150.2]	108.2 [90.5–123.5]	< 0.001
Right lung	74.6 [69.8–81.2]	59.2 [52.0–68.8]	< 0.001
Right upper lobe	30.4 [26.4–32.9]	25.2 [21.5–29.8]	< 0.001
Right middle lobe	13.6 [11.8–16.9]	13.4 [10.9–15.9]	0.225
Right lower lobe	31.7 [25.8–35.7]	20.2 [15.6–24.0]	< 0.001
Left lung	65.0 [56.9–71.6]	50.1 [41.8–58.8]	< 0.001
Left upper lobe	36.2 [33.4–39.2]	29.7 [24.8–35.1]	< 0.001
Left lower lobe	27.9 [24.1–33.7]	19.3 [15.3–23.6]	< 0.001
Upper lobes	66.1 [59.9–71.7]	52.2 [47.9–61.4]	< 0.001
Lower lobes	57.9 [51.2–67.1]	39.6 [32.4–49.0]	< 0.001

*3D-CT* 3- dimansional computed-tomography, *LV* lung volume, *IPF* idiopathic pulmonary fibrosis

**Fig. 2:**
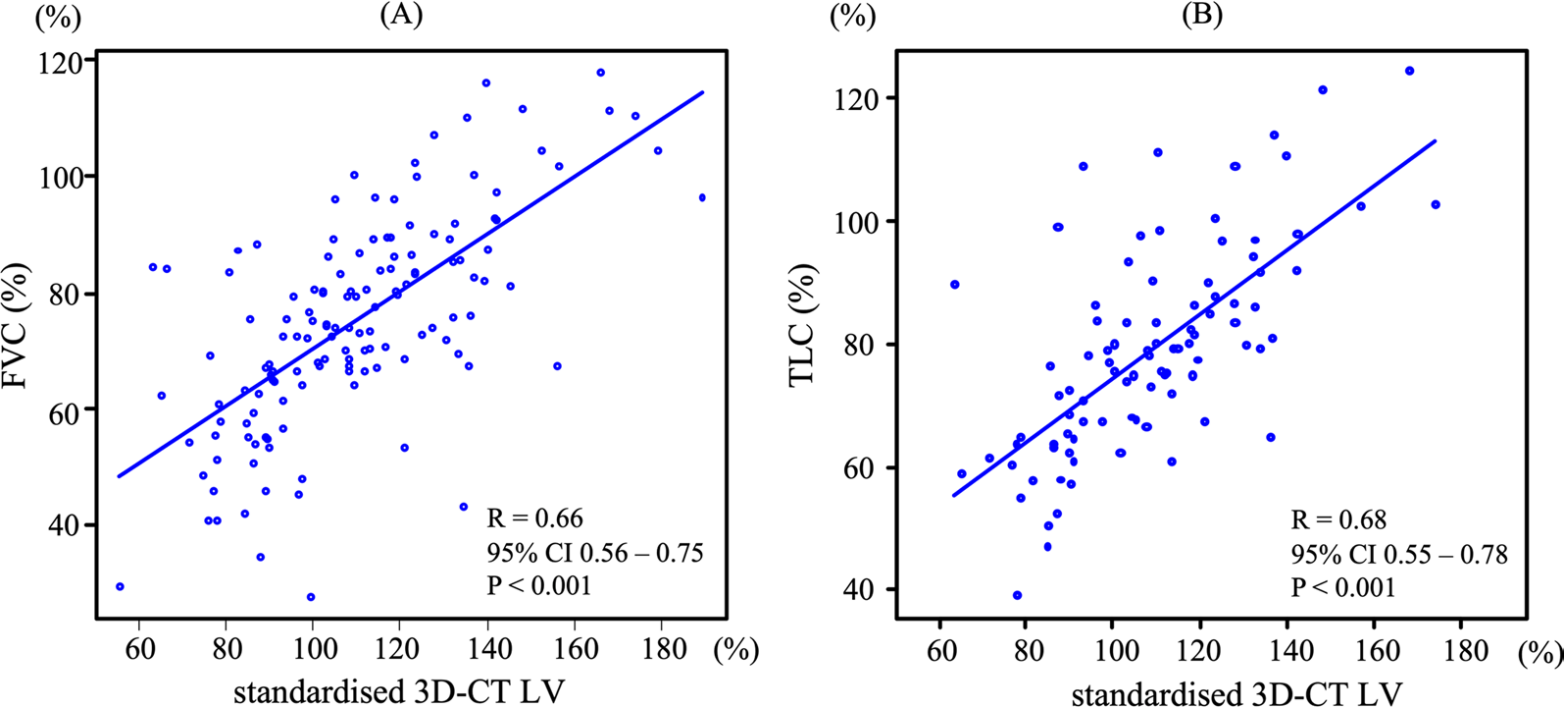
Correlations between the standardised three-dimensional lung volumes and forced vital capacity. **A** The correlation between the standardised three-dimensional computed tomography (3D-CT) lung volumes (LV) and forced vital capacity (FVC) is shown. **B** The correlation between the standardised 3D-CT LV (%) and FVC (%) is shown. The correlations were evaluated using the Pearson’s correlation coefficient

To validate the values of the standardised 3D-CT LV, correlation analyses were performed. The standardised 3D-CT LV (%) was found to be significantly correlated with FVC (%) (r = 0.66, p < 0.001), total lung capacity (TLC) (%) (r = 0.68, p < 0.001), and diffuse capacity of the lung for carbon monoxide (DLCO) (%) (r = 0.27, p = 0.009) (Fig. 2 and Additional file 1).

### Prognostic value of standardised 3D-CT LV in patients with IPF

**Fig. 3:**
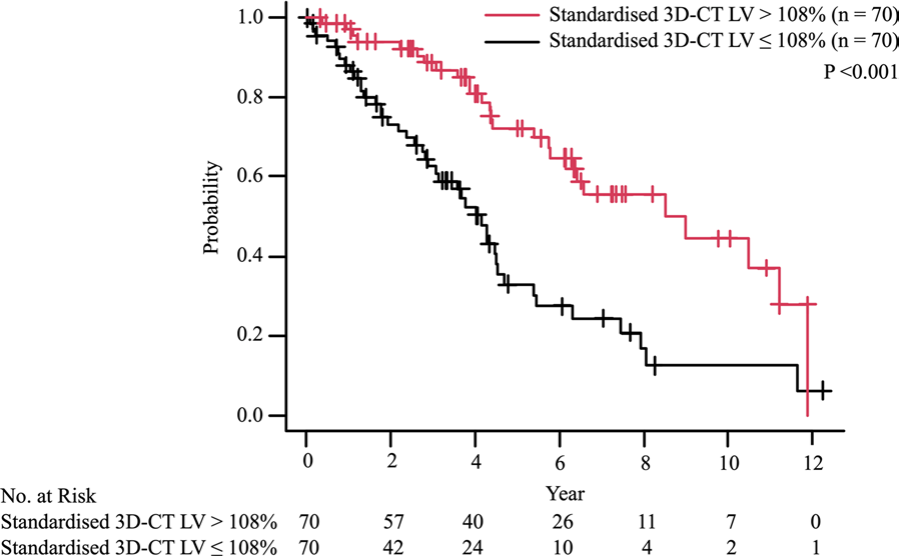
Prognostic impact of standardised three-dimensional computed tomography lung volumes at diagnosis. The Kaplan–Meier curves of patients with interstitial pulmonary fibrosis (IPF) based on the median value of the standardised three-dimensional computed tomography (3D-CT) lung volumes (LV) are shown. The cut-off value was 108%. The p-value was assessed using the log-rank test

We assessed the prognostic values of standardised 3D-CT LV at IPF diagnosis. A total of 69 patients died during the observation period. The median survival time was 5.47 years (IQR: 3.07–10.50 years). Patients with a standardised 3D-CT total LV > 108% had a significantly longer survival time than those with a standardised 3D-CT total LV < 108% (median survival time: 8.52 years vs. 4.16 years, p < 0.001) (Fig. 3).

The standardised 3D-CT LV (HR 0.976, p < 0.001), FVC (%) (HR 0.971, p < 0.001), FEV_1_/FVC (%) (HR 1.004, p = 0.025), DLCO (%) (HR 0.969, p = 0.003), PaO_2_ (HR 0.970, p < 0.001), KL-6 (HR 1.000, p < 0.001), and SP-D (HR 1.005, p = 0.001) were identified as prognostic factors for patients with IPF. When FVC (%) was excluded, the standardised 3D-CT LV at the time of IPF diagnosis was independently associated with mortality (HR 0.978, p = 0.002) (Additional file 2). The standardised 3D-CT LVs of the upper (HR 0.965, p = 0.001) and lower (HR 0.970, p = 0.005) lobes were also identified as prognostic factors (Additional file 2).

### Modified GAP system using standardised 3D-CT LV

The GAP system was well validated in determining the average risk of mortality of patients with IPF [13] and performed well in prognostic separation in our cohort. The median survival times of patients based on the GAP system were 7.47 years (IQR: 5.39–8.99 years) for patients classified as stage I, 4.35 years (IQR: 2.82–8.52 years) for patients classified as stage II, and 1.94 years (IQR: 0.21–2.64 years) for patients classified as stage III (Additional file 1). When standardised 3D-CT LV data were used in place of FVC data to determine the GAP stages, the median survival times were 7.47 years (IQR: 4.42–11.20 years) for patients classified as stage I, 4.27 years (IQR: 2.64–7.93 years) for patients classified as stage II, and 0.91 years for patients classified as stage III (IQR: 0.51–1.94 years) (Additional file 1). The modified GAP index showed similar discrimination performance as the original GAP index (C-statistics: 0.682 vs.0.658, respectively).

### Standardised 3D-CT LV in patients with AE of IPF

During the observation period, 61 AEs occurred in 47 patients with IPF, with a median time from IPF diagnosis of 5.90 months (IQR: 1.03–23.2 years). This study also enrolled another independent cohort patients with AE-IPF to validate values of the standardised 3D-CT LV (validation cohort). The patient characteristics at the time of AE are presented in Additional file 2. Representative segmental lung images obtained during AE are shown in Fig. 1C. The standardised 3D-CT LV for total lung and each lobe were significantly decreased compared to those obtained at the time of diagnosis (Table 3).

**Table 3:** Comparison of standardised 3D-CT LV in patients with IPF at diagnosis and at AE

Standardised 3D-CT LV, %	IPF at diagnosis (n = 61)	AE-IPF Hamamatsu cohort (n = 61)	AE-IPF Seirei cohort (n = 50)
Total lung	100.2 [85.3–114.0]	74.4 [66.5–84.5]*	75.4 [63.1–93.2]*
Right lung	55.6 [50.2–63.3]	43.6 [35.5–50.6]*	42.9 [35.9–51.5]*
Right upper lobe	25.0 [22.0–28.3]	18.5 [14.7–23.4]*	18.3 [15.6–22.9]*
Right middle lobe	12.3 [10.8–15.4]	10.2 [7.7–12.6]*	8.7 [7.0–10.7]*
Right lower lobe	17.9 [14.6–21.0]	13.4 [11.7–15.6]*	13.7 [11.6–15.9]*
Left lung	44.8 [36.1–50.5]	31.9 [25.4–37.1]*	32.1 [26.3–42.3]*
Left upper lobe	27.6 [23.6–34.1]	19.5 [16.0–22.0]*	18.7 [13.8–24.1]*
Left lower lobe	16.3 [12.4–20.8]	11.6 [9.6–14.1]*	14.2 [11.4–17.1]*
Upper lobes	51.9 [44.2–60.1]	38.4 [32.8–44.2]*	35.5 [28.6–48.2]*
Lower lobes	34.2 [26.9–41.1]	25.2 [21.3–29.7]*	26.6 [23.2–32.8]*

*3D-CT* 3-dimensional computed-tomography, *LV* lung volume, *IPF* idiopathic pulmonary fibrosis, *AE* acute enervation

*p < 0.001 (compared with that at IPF diagnosis)

### Prognostic value of standardised 3D-CT LV in patients with AE of IPF

**Fig. 4:**
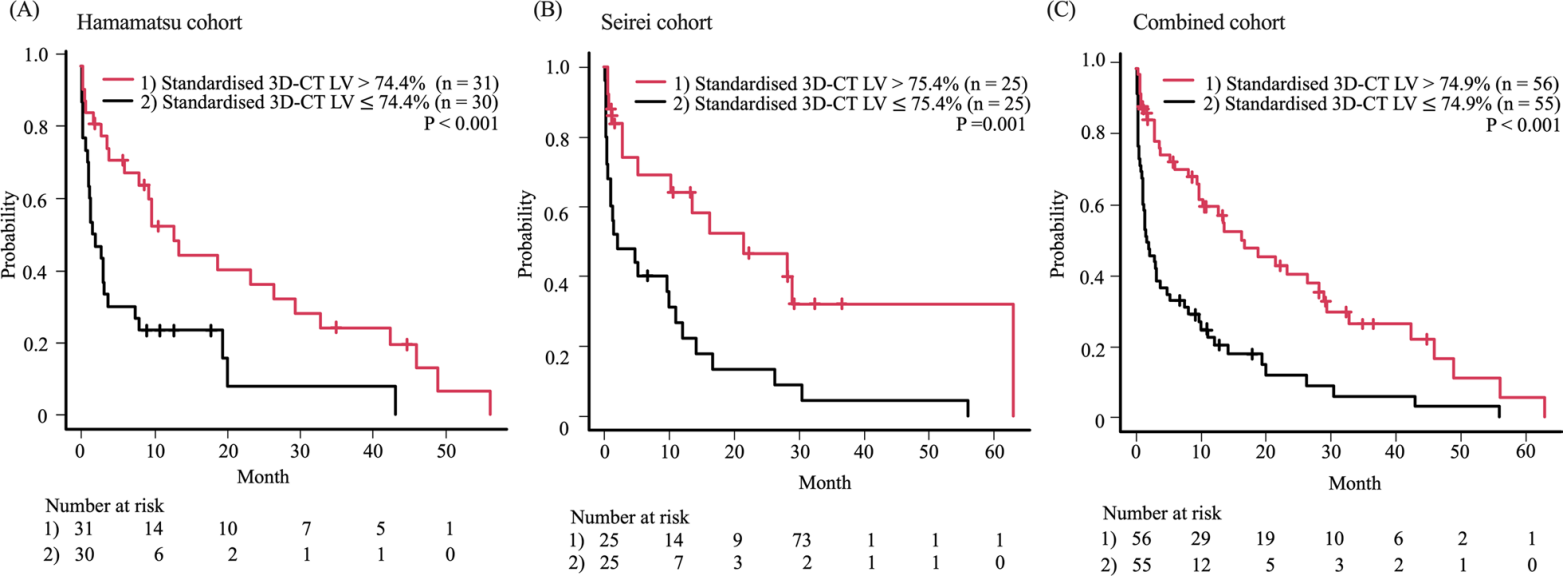
Prognostic impact of standardised three-dimensional computed tomography lung volumes at the time of acute exacerbation. The Kaplan–Meier curves of patients with interstitial pulmonary fibrosis based on the standardised three-dimensional computed tomography lung volumes at the time of acute exacerbation are shown; (**A**) Hamamatsu cohort, (**B**) Seirei cohort and (**C**) Combined cohort. A cut-off value of 74% was used. The p-value was assessed using the log-rank test

At the time of AE, PFTs are often difficult to perform due to the severe respiratory failure. However, CT can be taken with a light burden in such patients. Thus, we hypothesised that evaluating the standardised 3D-CT LV, instead of PFTs, would be beneficial in assessing pulmonary physiology in patients with AE of IPF. To justify the values, the cut-off values for standardised 3D-CT LV were determined by the median in each cohort. Patients with lower standardised 3D-CT LV had a significantly shorter survival than those with higher standardised 3D-CT (Hamamatsu cohort; median survival time: 1.73 months vs. 12.67 months, p $<$ 0.003, Seirei cohort; median survival time: 1.70 months vs. 16.60 months, p = 0.001, combined cohort; median survival time: 16.17 months vs. 1.53 months, p $<$ 0.001, Fig. 4).

The results of univariate and multivariate cox-proportional regression analyses in each cohort were shown in Additional file 2. The results showed that only standardised 3D-CT LV was consistently significant. In addition, the volume loss in the upper lobes, but not in the lower lobes, at the time of AE was identified as a prognostic factor (Additional file 2).

### Predictive model for the prognosis of patients with AE of IPF using standardised 3D-CT LV and CRP

**Fig. 5:**
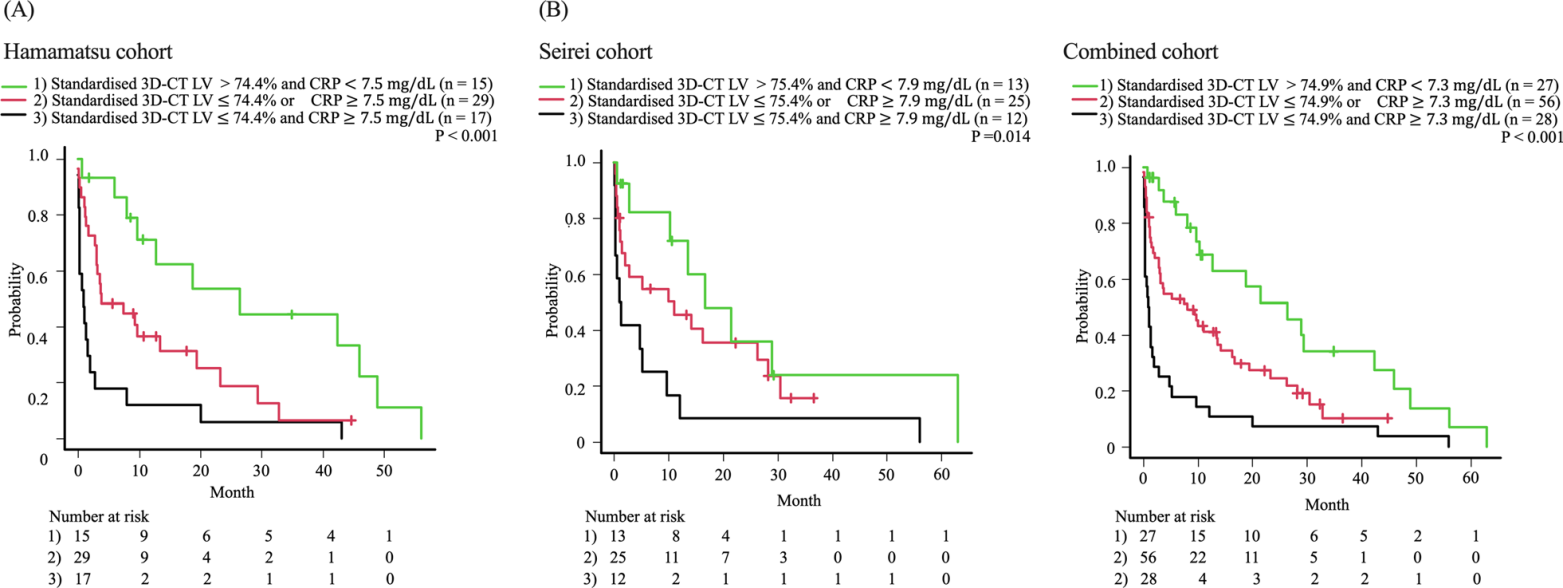
Standardised three-dimensional computed tomography lung volume and c-reactive protein to predict patient prognosis. The Kaplan–Meier curves of patients with interstitial pulmonary fibrosis according to the standardised three-dimensional computed tomography lung volumes and c-reactive protein (CRP) levels are shown; (**A**) Hamamatsu cohort, (**B**) Seirei cohort and (**C**) Combined cohort. The p-values were determined using the log-rank test

Given that standardised 3D-CT LV and CRP were proven to be significantly independent prognostic factors in patients with AE-IPF (combined cohort), we sought to develop a predictive model of prognosis for patients with AE-IPF using the standardised 3D-CT LV and CRP. Patients with AE of IPF were classified into three groups based on the median values of standardised 3D-CT LV and CRP in each cohort: (1) patients with above the median standardised 3D-CT LV and below the median CRP, (2) patients with below the median standardised 3D-CT LV and above the median CRP, and (3) remaining patients. The prognoses of these three groups were significantly different (p < 0.001, p = 0.014, and p < 0.001 respectively, Fig. 5). The C-statistics were 0.687, 0.651 and 0.678, respectively.

## Discussion

This study presents a novel quantification method for assessing pulmonary physiology using standardised 3D-CT-derived LVs. The standardised 3D-CT LV of the total lung was highly correlated with FVC (%) and was identified as an independent prognostic factor for patients with IPF. In addition, the modified GAP staging system using standardised 3D-CT LVs in place of FVCs effectively stratified patients with IPF based on mortality. Similarly, in patients with AE of IPF, the standardised 3D-CT LV of the total lung was an independent prognostic factor, and standardised 3D-CT LV can be combined with CRP data to predict the prognosis of patients with AE of IPF. Taken together, these results suggest that standardised 3D-CT LV accurately represents lung physiology and successfully predicts mortality in patients with IPF and patients with AE of IPF. As CT can be performed in patients with severe respiratory failure who cannot perform PFTs, standardised 3D-CT LV is a clinically-useful alternative to PFTs.

Several studies have previously found that 3D-CT-derived LVs are correlated with PFT parameters including FVC and TLC [15–18]. However, LVs are affected by age, sex, and body size; thus, standardising LVs with specific indicators is ideal. Therefore, a new image-quantification method is presented in this study. This method used the predicted FVC of each patient to determine the standardised 3D-CT LV (%). The standardised 3D-CT LV was closely correlated with FVC (%) and TLC (%) in patients with IPF, suggesting that standardised 3D-CT LVs accurately represent FVC (%) and TLC (%) in patients with IPF.

The cardinal feature of IPF is progressive lung fibrosis that results in respiratory failure and decreased lung capacity [1, 2]. PFTs can be used to assess disease severity and progression and are essential for predicting outcomes in patients with IPF [13, 19–29]. However, in patients with advanced IPF or AEs of IPF, PFTs cannot be performed accurately due to severe respiratory failure. For these patients, 3D-CT imaging is much less burdensome than PFTs. The results of this study suggest that patients with IPF with higher standardised 3D-CT LV (> 108%) at the time of diagnosis had a significantly longer survival than those with lower standardised 3D-CT LV (≤ 108%). Similarly, patients with higher standardised 3D-CT LV at AE had a significantly longer survival than those with lower standardised 3D-CT LV at AE. In addition, standardised 3D-CT LV was identified as an independent prognostic factor in patients with IPF and in those with AE of IPF. Moreover, the modified GAP system (in which standardised 3D-CT LVs were used instead of FVCs (%)) accurately predicted patient mortality. Standardised 3D-CT LV during AE was also identified as a predictor of mortality. These results suggest that standardised 3D-CT LV provides valuable information regarding disease progression and prognosis, especially in patients with IPF with severe respiratory failure who cannot perform PFTs.

The 3D-CT LVs of each lung lobe provides additional insight into the pathophysiology of IPF and AE of IPF. The standardised 3D-CT LV of the total lung and each lobe at the time of diagnosis were significantly reduced in patients with IPF compared with control subjects (with the exception of the middle lung lobe). Furthermore, the decrease was more prominent in the lower lobes than in the upper lobes. However, the prognostic significance of the upper and lower lobes was comparable (Additional file 2). In contrast, a remarkable decrease in the standardised 3D-CT LV during AE was observed in all lobes. Similar to the 3D-CT images at the time of IPF diagnosis, the LVs of the lower lobes decreased more than those of the upper lobes in patients with AE. However, striking difference was noted in the prognostic significance of the lobes between the 3D-CT images at IPF diagnosis and those at AE (Additional file 2). Interestingly, in patients with AE of IPF, the standardised 3D-CT LV of the lower lobes had no prognostic impact, while the standardised 3D-CT LV of the upper lobes was significantly associated with survival (Additional file 2). In patients with AE of IPF, diffuse ground glass opacities and/or consolidations were found to be newly superimposed on underlying fibrotic changes located predominantly in the lower lobes. These new opacities can cause severe respiratory failure as they are considered to be located in the remaining normal lung structures of patients with IPF. As the upper lobes generally have normal or mild lesions in patients with IPF, the involvement of the upper lobes in AE may be more strongly associated with mortality than the involvement of the lower lobes. In contrast, the LV of the lower lobes during AE may not affect survival as fibrotic changes were present in these lobes prior to AE. Therefore, upper lobe lesions during AE of IPF are believed to be more prognostic than lower lung lesions.

This study has some limitations, including its retrospective, single-cohort design and the lack of participation on behalf of experienced radiologists. In addition, patients with a history of lung resection were excluded from this study. The usefulness of standardised 3D-CT in these patients should be assessed in a future study. Furthermore, the CT images may not have been obtained properly in some patients as the patients were required to be at full inspiration, which may not have been feasible in patients with severe respiratory failure.

## Conclusions

Standardised lung volumes derived from 3D-CT images accurately represent lung physiology in patients with IPF and can be used alone or combined with other indicators to predict survival among these patient populations. Standardised 3D-CT LV is a useful alternative to PFTs in patients who cannot undergo PFTs due to severe respiratory failure.

## Supplementary Information

**Additional file 1.** Supplementary Figures.

**Additional file 2.** Supplementary Tables.

## Data Availability

The data and materials that support the findings of this study are available from the corresponding author upon reasonable request.

## Abbreviations

3D-CT: 3-Dimensional computed-tomography
AE: Acute exacerbation
AFT: Antifibrotic therapy
BMI: Body mass index
CRP: C-reactive protein
DLCO: Diffusing capacity of the lung for carbon monoxide
FEV1.0: Forced expiratory volume in 1.0 s
FVC: Forced vital capacity
GAP index: Gender-age-physiology index
ILDs: Interstitial lung diseases
IPF: Idiopathic pulmonary fibrosis
KL-6: Krebs von den Lungen-6
LV: Lung volume
PFTs: Pulmonary function tests
SP-D: Surfactant protein-D
UIP: Usual interstitial pneumonia
