# Supplementary material for: Standardised 3D-CT lung volumes for patients with idiopathic pulmonary fibrosis
Source: Respir Res. 2022 Jun 1;23:142. doi: 10.1186/s12931-022-02062-1 (PMC9161591; doi:10.1186/s12931-022-02062-1)
Supplement: Supplementary file 1 — Additional file 1. Supplementary Figures. [file 12931_2022_2062_MOESM1_ESM.docx]

**Supplementary Figures**


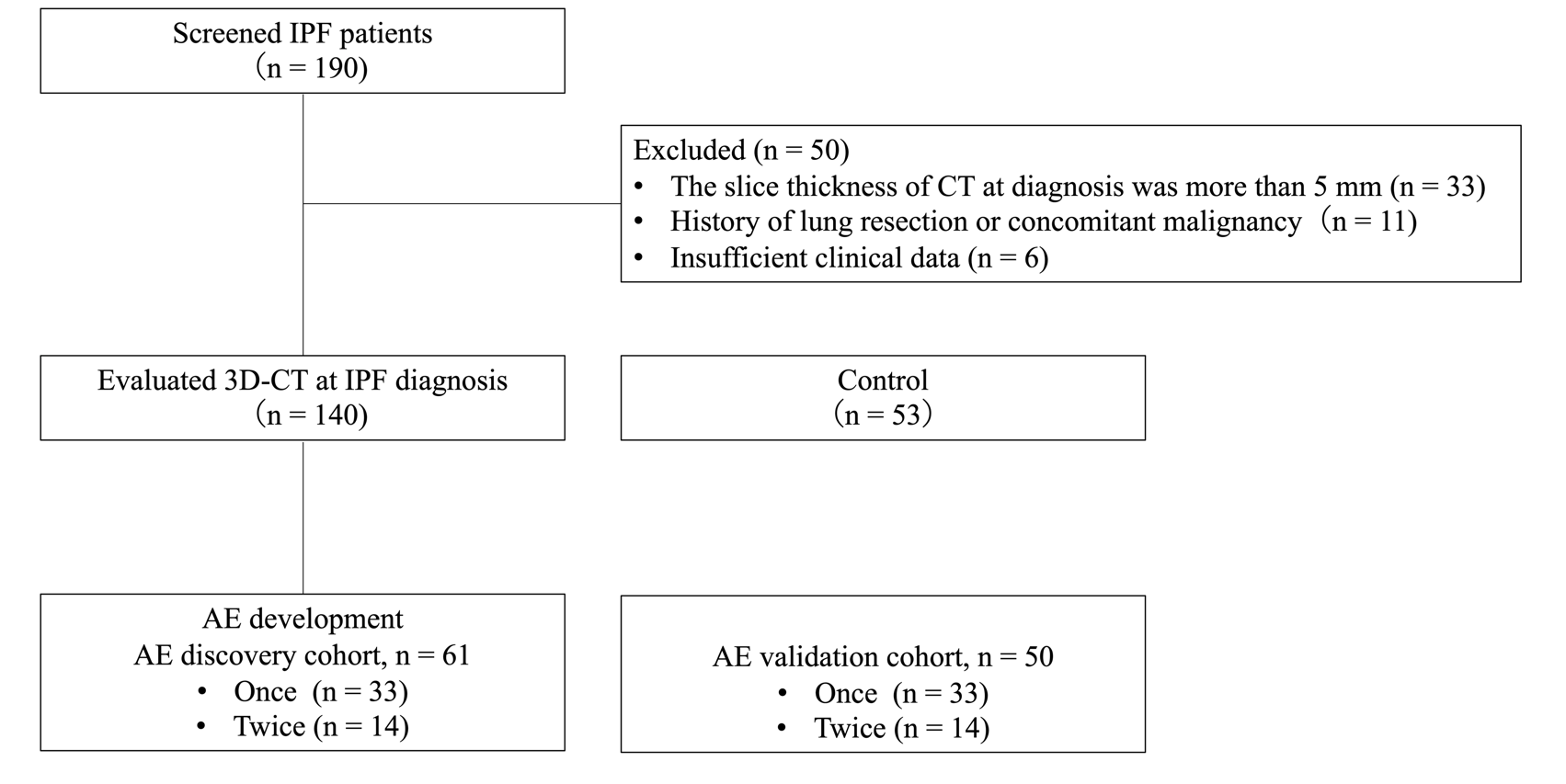
**Supplementary Figure 1. Flow chart**

**Supplementary Figure 2. Correlations between the standardised three-dimensional lung volumes and diffuse capacity of the lung for carbon monoxide**


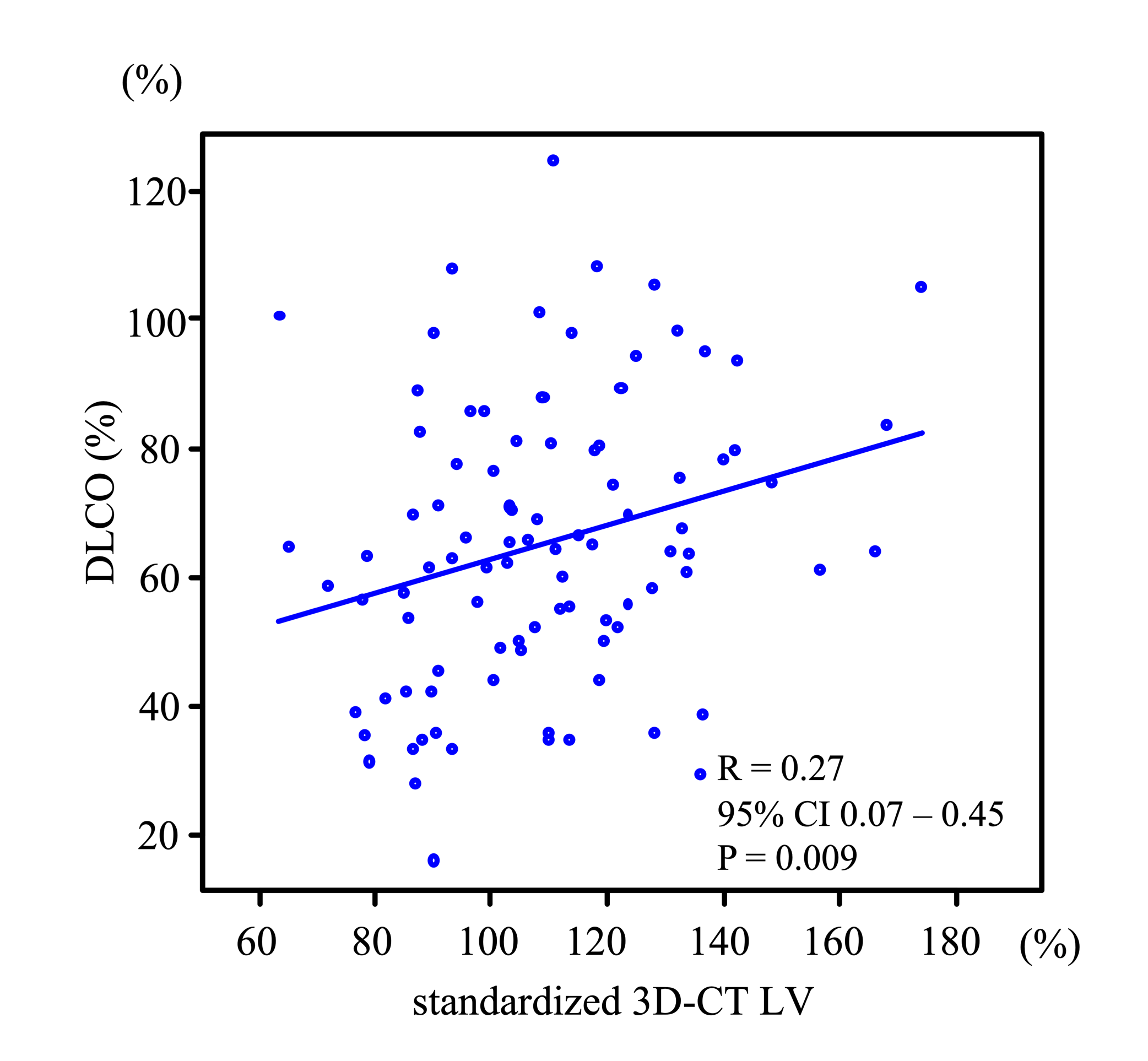
The correlation between the three-dimensional computed tomography lung volumes and diffuse capacity of the lung for carbon monoxide. The correlations were assessed using the Pearson's correlation coefficient.


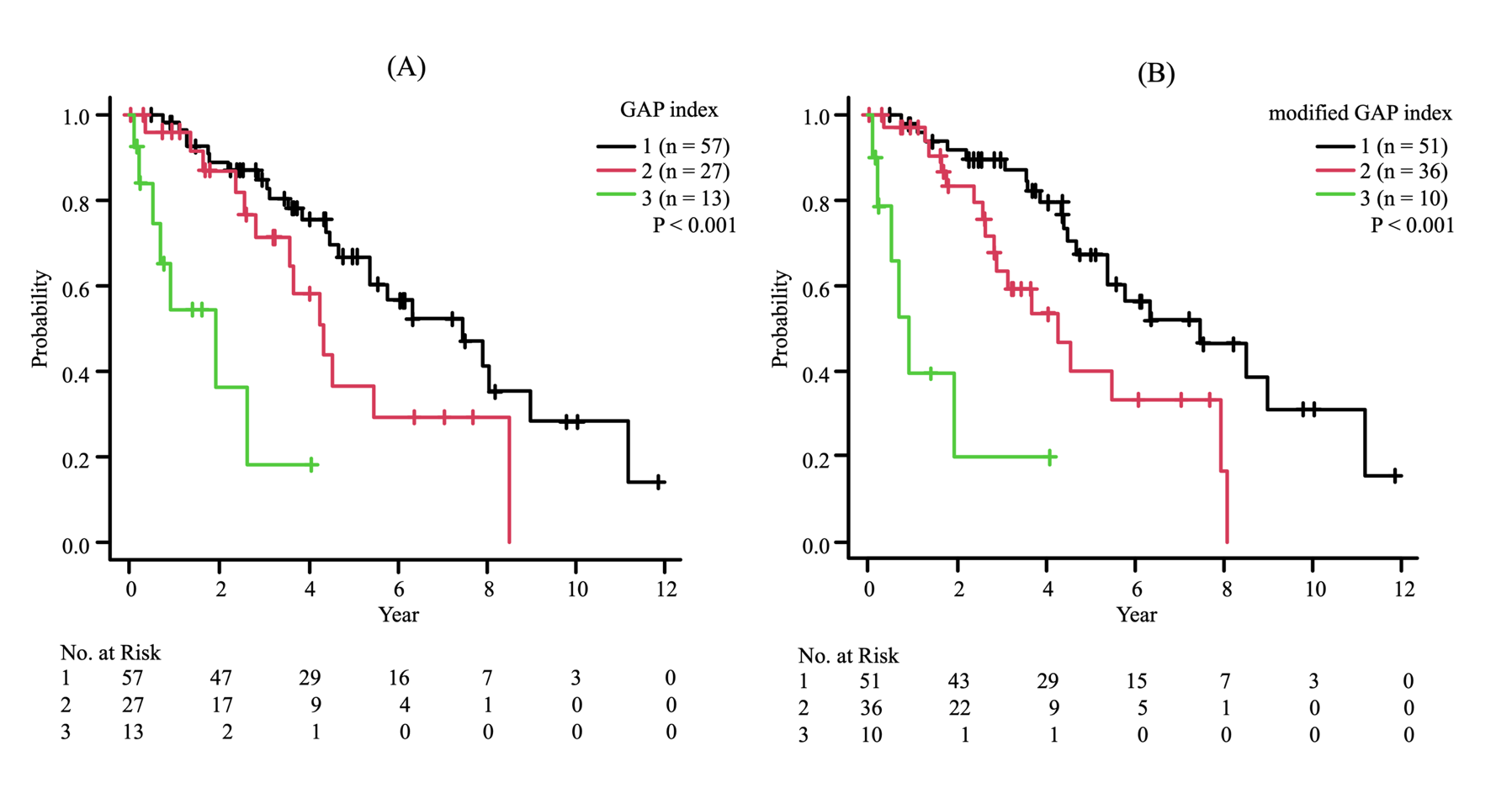
**Supplementary Figure 3. Patient prognosis according to the Gender-Age-Physiology index and modified Gender-Age-Physiology index**

A. The Kaplan–Meier curves of patients with interstitial pulmonary fibrosis (IPF) based on the Gender-Age-Physiology (GAP) index at the time of diagnosis are represented. B. The Kaplan–Meier curves of patients with IPF based on the modified GAP index are shown. The modified GAP index was calculated using standardised three-dimensional computed tomography lung volumes in place of forced vital capacity. The p-value was assessed using the log-rank test.
