# Supplementary material for: Standardised 3D-CT lung volumes for patients with idiopathic pulmonary fibrosis
Source: Respir Res. 2022 Jun 1;23:142. doi: 10.1186/s12931-022-02062-1 (PMC9161591; doi:10.1186/s12931-022-02062-1)
Supplement: Supplementary file 2 — Additional file 2. Supplementary Tables. [file 12931_2022_2062_MOESM2_ESM.docx]

***Supplementary Tables***

***Supplementary Table 1. Prediction of mortality in patients with IPF at diagnosis by univariate and multivariate Cox-proportion analyses***

| **Predictor** | **HR** | **95% CI** | **p-value** |  | **HR** | **95% CI** | **p-value** |
| --- | --- | --- | --- | --- | --- | --- | --- |
| **Univariate analysis** |  |  |  | **Multivariate analysis 1** |  |  |  |
| Age, year | 1.032 | 0.999–1.066 | 0.054 | Age, year | 1.021 | 0.983–1.061 | 0.283 |
| Sex, male | 0.698 | 0.364–1.340 | 0.280 | Sex, male | 0.712 | 0.364–1.392 | 0.321 |
| BMI, kg/m^2^ | 1.002 | 0.924–1.087 | 0.965 | PaO_2_, Torr | 0.980 | 0.961–1.000 | 0.053 |
| FVC, percent predicted | 0.971 | 0.956–0.985 | <0.001 | KL-6, U/ml | 1.000 | 1.000–1.001 | 0.014 |
| FEV_1_/FVC, % | 1.044 | 1.006–1.083 | 0.023 | SP-D, ng/ml | 1.001 | 1.000–1.003 | 0.156 |
| DLCO, % | 0.969 | 0.949–0.999 | 0.003 | Standardized 3D-CT LV, % | 0.978 | 0.964–0.991 | 0.001 |
| PaO_2_, Torr | 0.970 | 0.954–0.986 | <0.001 |  |  |  |  |
| KL-6, U/ml | 1.000 | 1.000–1.001 | <0.001 |  |  |  |  |
| SP-D, ng/ml | 1.005 | 1.002–1.008 | <0.001 | **Multivariate analysis 2** |  |  |  |
| CRP, mg/dl | 0.918 | 0.576–1.464 | 0.720 | Age, year | 1.010 | 0.973–1.048 | 0.606 |
| Standardized 3D-CT LV, % | 0.976 | 0.965–0.988 | <0.001 | Sex, male | 0.770 | 0.392–1.514 | 0.449 |
|  |  |  |  | PaO_2_, Torr | 0.973 | 0.955–0.992 | 0.005 |
|  |  |  |  | KL-6, U/ml | 1.000 | 1.000–1.001 | 0.029 |
|  |  |  |  | SP-D, ng/ml | 1.001 | 1.000–1.003 | 0.100 |
|  |  |  |  | FVC, percent predicted | 0.976 | 0.960–0.992 | 0.005 |

IPF; idiopathic pulmonary fibrosis, BMI; body mass index, FVC; forced vital capacity, FEV_1.0_; forced expiratory volume in 1.0 second, DLCO; diffuse capacity of the lung for carbon monoxide, P/F ratio; PaO_2_ / FiO_2_ ratio, KL-6; Krebs von den Lunge-6, SP-D; surfactant protein-D, CRP; C-reactive protein, 3D-CT; 3-dimansional computed-tomography, LV; lung volume, HR; hazard ratio, CI; confidence interval

***Supplementary Table 2. Capacity of lung lobes for predicting mortality in patients***

| **Predictor** | **IPF at diagnosis (n = 140)** | | | **AE-IPF (n = 61)**  **Hamamatsu cohort** | | | **AE-IPF (n = 50)**  **Seirei cohort** | | | |
| --- | --- | --- | --- | --- | --- | --- | --- | --- | --- | --- |
| **Age and gender-adjusted**  **standardised 3D-CT LV, %**  **univariate analysis** | **HR** | **95% CI** | **p-value** | **HR** | **95% CI** | **p-value** | | **HR** | **95% CI** | **p-value** |
| Total lung | 0.975 | 0.963–0.987 | <0.001 | 0.969 | 0.951–0.988 | 0.001 | | 0.969 | 0.949–0.988 | 0.002 |
| Right lung | 0.958 | 0.938–0.978 | <0.001 | 0.942 | 0.914–0.971 | <0.001 | | 0.964 | 0.928–1.001 | 0.057 |
| Right upper lobe | 0.968 | 0.932–1.004 | 0.081 | 0.914 | 0.861–0.971 | 0.004 | | 0.927 | 0.878–0.979 | 0.006 |
| Right middle lobe | 0.891 | 0.832–0.955 | 0.001 | 0.902 | 0.836–0.972 | 0.007 | | 0.821 | 0.717–0.941 | 0.005 |
| Right lower lobe | 0.960 | 0.929–0.990 | 0.011 | 0.996 | 0.959–1.035 | 0.823 | | 1.020 | 0.969–1.074 | 0.448 |
| Left lung | 0.961 | 0.941–0.981 | <0.001 | 0.969 | 0.935–1.004 | 0.079 | | 0.942 | 0.909–0.976 | <0.001 |
| Left upper lobe | 0.945 | 0.917–0.973 | <0.001 | 0.954 | 0.904–1.007 | 0.089 | | 0.917 | 0.873–0.962 | <0.001 |
| Left lower lobe | 0.948 | 0.909–0.988 | 0.012 | 0.954 | 0.880–1.033 | 0.246 | | 0.931 | 0.854–1.016 | 0.107 |
| Upper lobe | 0.965 | 0.962–0.985 | <0.001 | 0.958 | 0.927–0.989 | 0.008 | | 0.946 | 0.916–0.976 | <0.001 |
| Lower lobe | 0.970 | 0.950–0.991 | 0.005 | 0.989 | 0.955–1.024 | 0.522 | | 0.992 | 0.948–1.037 | 0.514 |

3D-CT; 3-dimansional computed-tomography, LV; lung volume, IPF; idiopathic pulmonary fibrosis, AE; Acute enervation, HR; hazard ratio, CI; confidence interval

***Supplementary Table 3. Clinical characteristics of patients with AE-IPF***

|  | **AE-IPF**  **Hamamatsu cohort (n = 61)** | **AE-IPF**  **Seirei cohort (n = 50)** |
| --- | --- | --- |
| Age, year | 74 [69–78] | 77 [71-84] |
| Sex, male/female | 49 (80.3%) / 12 (19.7%) | 53 (88.3%) / 7 (11.7%) |
| cIPF / UIP/IPF | 48 (78.7%) / 13 (21.3%) | 48 (96.0%) / 2 (4.0%) |
| Time to diagnosis to AE, years | 1.9 [0.9–3.4] | 0.42 [0.08–1.35] |
| Never smoker | 11 (18.0%) | 9 (18.0%) |
| Former or current smoker | 50 (82.0%) | 41(82.0%) |
| Height, cm | 161.0 [155.0–164.0] | 161.5 [155.6–165.0] |
| Weight, kg | 56.3 [50.4–61.6] | 56.5 [48.4–61.4] |
| BMI, kg/m^2^ | 21.9 [20.5–23.6] | 22.1 [19.3–24.2] |
| Laboratory |  |  |
| P/F ratio | 209.5 [137.5–281.0] | 232.2 [158.4-280.0] |
| KL-6, U/ml | 1531 [1038–2232] (n = 59) | 1602 [1007–2165] |
| SP-D, ng/ml | 333 [227–524] (n = 57) | 297 [182–537] |
| CRP, mg/dL | 5.54 [2.54–9.92] (n = 59) | 7.85 [4.25–12.28] |
| Treatment |  |  |
| Pirfenidone / Nintedanib | 28 (45.9%) / 2 (3.3%) | 13(26%) / 8 (16%) |

IPF; idiopathic pulmonary fibrosis, AE; acute exacerbation, UIP; usual interstitial pneumonia, BMI; body mass index, FVC; forced vital capacity, FEV_1.0_; forced expiratory volume in 1.0 second, DLCO; diffuse capacity of the lung for carbon monoxide, GAP; Gender-Age-Physiology, KL-6; Krebs von den Lunge-6, SP-D; surfactant protein-D, CRP; C-reactive protein

***Supplementary Table 4. Prediction of mortality in patients with AE-IPF by multivariate Cox-proportion analyses***

|  | **AE-IPF**  **Hamamatsu cohort (n=61)** | | | **AE-IPF**  **Seirei cohort (n=50)** | | | **AE-IPF**  **Combined cohort (n=111)** | | |
| --- | --- | --- | --- | --- | --- | --- | --- | --- | --- |
| **Predictor** | **HR** | **95% CI** | **p-value** | **HR** | **95% CI** | **p-value** | **HR** | **95% CI** | **p-value** |
| **Multivariate analyses** |  |  |  |  |  |  |  |  |  |
| Age, year | 1.013 | 0.961–1.068 | 0.620 | 1.008 | 0.956 – 1.062 | 0.779 | 1.003 | 0.969 – 1.038 | 0.869 |
| Sex, male | 1.103 | 0.451–2.949 | 0.837 | 0.876 | 0.339 – 2.724 | 0.800 | 0.901 | 0.490 – 1.749 | 0.747 |
| P/F ratio | 1.000 | 0.996–1.005 | 0.829 | 1.001 | 0.997 – 1.006 | 0.501 | 0.999 | 0.997 – 1.003 | 0.978 |
| CRP, mg/dl | 1.107 | 1.036–1.184 | 0.004 | 1.062 | 1.004 – 1.118 | 0.028 | 1.068 | 1.026 – 1.109 | <0.001 |
| standardised 3D-CT LV, % | 0.964 | 0.953–0.984 | <0.001 | 0.962 | 0.938 – 0.982 | <0.001 | 0.968 | 0.954 – 0.981 | <0.001 |

AE; acute exacerbation, IPF; idiopathic pulmonary fibrosis, BMI; body mass index, P/F ratio; PaO_2_ / FiO_2_ ratio, AFT; antifibrotic therapy, KL-6; Krebs von den Lunge-6, SP-D; surfactant protein-D, CRP; C-reactive protein, 3D-CT; 3-dimansional computed-tomography, LV; lung volume, HR; hazard ratio, CI; confidence interval

***Supplementary Table 5. Prediction of mortality in patients with AE-IPF in the combined cohort by univariate Cox-proportion analyses***

|  | **AE-IPF**  **Hamamatsu cohort (n=61)** | | | **AE-IPF**  **Seirei cohort (n=50)** | | | **AE-IPF**  **Combined cohort (n=111)** | | |
| --- | --- | --- | --- | --- | --- | --- | --- | --- | --- |
| **Predictor** | **HR** | **95% CI** | **p-value** | **HR** | **95% CI** | **p-value** | **HR** | **95% CI** | **p-value** |
| Age, year | 1.010 | 0.969–1.052 | 0.645 | 0.986 | 0.943 – 1.030 | 0.526 | 0.996 | 0.967 – 1.038 | 0.629 |
| Sex, male | 1.396 | 0.673–2.897 | 0.370 | 0.779 | 0.329 – 2.290 | 0.606 | 0.872 | 0.497 – 1.530 | 0.634 |
| BMI, kg/m^2^ | 0.955 | 0.868–1.051 | 0.347 | 0.960 | 0.865 – 1.066 | 0.445 | 0.969 | 0.905 – 1.038 | 0.366 |
| P/F ratio | 0.997 | 0.994–1.000 | 0.045 | 0.999 | 0.996 – 1.003 | 0.776 | 0.998 | 0.996 – 0.999 | 0.069 |
| AFT (Pirfenidone or Nintedanib) | 1.044 | 0.589–1.852 | 0.883 | 1.904 | 0.947 – 3.830 | 0.071 | 1.324 | 0.855 – 2.053 | 0.209 |
| KL-6, U/ml | 1.000 | 1.000–1.000 | 0.588 | 1.000 | 1.000 – 1.000 | 0.835 | 1.000 | 1.000 – 1.000 | 0.628 |
| SP-D, ng/ml | 1.000 | 0.999–1.001 | 0.636 | 1.000 | 1.00 – 1.001 | 0.969 | 1.000 | 0.999 – 1.001 | 0.735 |
| CRP, mg/dl | 1.063 | 1.015–1.113 | 0.010 | 1.026 | 0.975 – 1.080 | 0.322 | 1.041 | 1.005 – 1.075 | 0.020 |
| standardised 3D-CT LV, % | 0.973 | 0.955–0.991 | 0.003 | 0.970 | 0.952 – 0.988 | <0.001 | 0.972 | 0.960 – 0.985 | <0.001 |

AE; acute exacerbation, IPF; idiopathic pulmonary fibrosis, BMI; body mass index, P/F ratio; PaO_2_ / FiO_2_ ratio, AFT; antifibrotic therapy, KL-6; Krebs von den Lunge-6, SP-D; surfactant protein-D, CRP; C-reactive protein, 3D-CT; 3-dimansional computed-tomography, LV; lung volume, HR; hazard ratio, CI; confidence interval
